# Accuracy and Limitations of Sentinel Lymph Node Biopsy after Neoadjuvant Chemotherapy in Breast Cancer Patients with Positive Nodes

**DOI:** 10.1155/2022/1507881

**Published:** 2022-08-05

**Authors:** Sofia Aragon-Sanchez, M. Reyes Oliver-Perez, Ainhoa Madariaga, M. Jose Tabuenca, Mario Martinez, Alberto Galindo, M. Luisa Arroyo, Marta Gallego, Marta Blanco, Eva M. Ciruelos-Gil

**Affiliations:** ^1^Breast Cancer Unit, Department of Obstetrics and Gynaecology, University Hospital 12 de Octubre, Madrid, Spain; ^2^Research Institute (imas12), Complutense University of Madrid, Madrid, Spain; ^3^Gynaecology Oncology Unit, Department of Obstetrics and Gynaecology, University Hospital 12 de Octubre, Madrid, Spain; ^4^Breast and Gynaecology Cancer Unit, Department of Medical Oncology, University Hospital 12 de Octubre, Madrid, Spain; ^5^Department of Nuclear Medicine, University Hospital 12 de Octubre, Madrid, Spain; ^6^Department of Pathology, University Hospital 12 de Octubre, Madrid, Spain; ^7^Fetal Medicine Unit- Maternal and Child Health and Development Network (Red SAMIDRD 12/0026/0016), Department of Obstetrics and Gynaecology, University Hospital 12 de Octubre, Madrid, Spain

## Abstract

**Background:**

Axillary surgical management in patients with node-positive breast cancer at the time of diagnosis converted to negative nodes through neoadjuvant chemotherapy (NAC) remains unclear. Removal of more than two sentinel nodes (SLNs) in these patients may decrease the false negative rate (FNR) of sentinel lymph node biopsies (SLNBs). We aim to analyse the detection rate (DR) and the FNR of SLNB assessment according to the number of SLNs removed.

**Methods:**

A retrospective study was performed from October 2012 to December 2018. Patients with invasive breast cancer who had a clinically node-positive disease at diagnosis and with a complete axillary response after neoadjuvant chemotherapy were selected. Patients included underwent SLNB and axillary lymph node dissection (ALND) after NAC. The SLN was considered positive if any residual disease was detected. Descriptive statistics were used to describe the clinicopathologic features and the results of SLNB and ALND. The DR of SLNB was defined as the number of patients with successful identification of SLN. Presence of residual disease in ALND and negative SLN was considered false negative.

**Results:**

A total of 368 patients with invasive breast cancer who underwent surgery after complete NAC were studied. Of them, 85 patients met the eligibility criteria and were enrolled in the study. The mean age at diagnosis was 50.8 years. Systematic lymphadenectomy was performed in all patients, with an average of 10 lymph nodes removed. The DR of SLNB was 92.9%, and the FNR was 19.1. The median number of SLNs removed was 3, and at least, three SLNs were obtained in 42 patients (53.2%). When at least three sentinel nodes were removed, the FNR decreased to 8.7%.

**Conclusions:**

In this cohort, the SLN assessment was associated with an adequate DR and a high FNR. Removing three or more SLNs decreased the FNR from 19.1% to 8.7%. Complementary approaches may be considered for axillary lymph node staging after neoadjuvant chemotherapy. The study was approved by our institution's ethics committee (Instituto de Investigacion Sanitaria Hospital 12 de Octubre (imas12), Universidad Complutense de Madrid, Madrid, Spain) (https://clinicaltrials.gov/ct2/show/NCEI:20/0048).

## 1. Introduction

Axillary staging in patients with breast cancer has experienced significant changes over the last two decades. Sentinel lymph node biopsy (SLNB) has replaced axillary lymph node dissection (ALND) in clinically node-negative breast cancer patients, reporting similar disease-free survival and overall survival rates with fewer side effects [1–3]. Nonetheless, in patients with clinically positive nodes converted to negative through neoadjuvant chemotherapy (NAC), optimal axillary management remains unclear.

In this setting, the first prospective trials and subsequent meta-analysis reported higher false negative rates (FNRs) than the accepted cutoff value of 10% [4–6]. Several approaches have been proposed to improve axillary staging after NAC such as the removal of at least three negative sentinel lymph nodes (SLNs), the use of dual tracers, pathological staining by immunochemistry when SLNs are negative, and selection of patients with a clinical axillary response after completion NAC [4, 5, 7, 8]. More recently, targeted axillary dissection (TAD), which includes selective removal of metastatic lymph nodes marked before neoadjuvant therapy and SLNB, and tailored axillary surgery (TAS), designed to reduce the tumour load in the axilla by performing SLNB and palpation-guided selective removal of suspicious nodes, have shown a FNR lower than 5%. [9–12]. As a result, there is a wide heterogeneity of recommendations endorsed by different international guidelines and societies about the most adequate management of node-positive patients converted to negative through NAC. Moreover, to date, data on its impact on disease-free and overall survival are lacking [13].

The primary objective of this study was to analyse the accuracy of SLNB after NAC in patients who were initially clinically node positive and converted to negative through treatment. In addition, we aimed to determine the impact of the number of SNLs removed on the detection rate and FNR.

## 2. Materials and Methods

This was a retrospective study. We selected women with primary invasive breast cancer, clinical stage T1-3 and N1 in the pretreatment evaluation, who underwent surgery after receiving NAC from October 2012 to December 2018 at the Breast Cancer Unit of Hospital 12 de Octubre. Clinically node-negative (cN0) patients prior to NAC, those without a complete axillary response after NAC, and those who, according to our local guidelines, did not undergo SLNB because of clinical stage T4 and/or cN2 were excluded. Only patients in whom the SLN was analysed by haematoxylin and eosin (H&E) were included.

The study was approved by our institution's ethics committee (Instituto de Investigacion Sanitaria Hospital 12 de Octubre (imas12), Universidad Complutense de Madrid, Madrid, Spain) (NCEI: 20/0048), which waived the informed consent from the patients due to the retrospective nature of the study.

### 2.1. Pretreatment Evaluation

Breast evaluation was performed by physical exam, mammography, ultrasound, and magnetic resonance imaging. Diagnosis of invasive breast carcinoma was confirmed by image-guided core needle biopsy, and a radiopaque clip was placed. All histologic exams were performed by breast cancer-specialized pathologists. Histologic classification and architectural grade were performed in accordance with the Nottingham Histologic Score [14]. Oestrogen and progesterone receptor expression, HER- 2 status, and the tumour proliferation index (Ki-67) were noted as per the American Society of Clinical Oncology/College of American Pathologists guidelines [15]. Tumour biological phenotypes were classified based on the Goldhirsch and Viale criteria [16, 17].

Axillary staging was performed by physical exam and axillary ultrasound. The most suspicious node according to clinical or morphologic criteria was confirmed by fine needle aspiration in all patients before treatment [18]. Tumour staging was classified based on the seventh edition of the American Joint Committee on Cancer [19]. Tumour size was determined as the largest of the three tumour measurements on magnetic resonance imaging in all patients.

### 2.2. Neoadjuvant Treatment

NAC was decided in our weekly multidisciplinary tumour committee according to specific evidence-based guidelines and individual medical features. We have internal protocols for the use of neoadjuvant systemic therapies in accordance with national and international guidelines for clinical practice [20–23]. For HER2^+^ patients with axillary involvement, an anthracycline-based regimen was considered, such as anthracycline/cyclophosphamide (AC) *x* 4 cycles, followed by paclitaxel trastuzumab and pertuzumab. In triple-negative disease, nab-paclitaxel plus carboplatin *x* 4 cycles followed by an AC *x* 4 regimen was considered. In luminal phenotypes, a combination of ACx4 followed by weekly paclitaxel *x* 12 was considered.

### 2.3. Posttreatment Evaluation and Management

The response at the breast was assessed by physical exam and magnetic resonance imaging. The axillary response after NAC was determined by physical examination and axillary ultrasound.

The surgery was performed three or five weeks after finishing NAC by a specialist in breast cancer surgery.

Breast-conserving surgery consisted in lumpectomy with oncoplastic procedures if necessary to ensure cosmetic outcomes. Free margins were considered if the ink was negative. An skin-sparing mastectomy with reconstruction was considered in the following cases: inadequate cosmetic result with conservative approach and/or hereditary breast cancer.

All patients underwent SNB and ALND. The agent used for lymphoscintigraphy and SLN localization was (99 mTc) Tc-nanocolloidal-albumin injected intradermally and in the subareolar region either on the day of surgery or the day before. A handheld gamma probe was used to identify the maximum radioactivity in the axilla. All lymph nodes with a radioactive count above 10% of the ex vivo counts measured in the hottest SLN were removed. No other colorimetric agents were used to search SLNs in case of no migration of the radiotracer.

Pathologic examination of SLN was performed by H&E staining. SLN evaluation was deferred, not performed intraoperatively, to paraffin sections separated by 3 *μ*m and stained with cytokeratin immunohistochemistry at each one when H&E was negative. The SLN was considered positive when isolated tumour cells, micrometastases, or macrometastases were detected. ALND was analysed by H&E staining.

### 2.4. Statistical Analysis

Categorical variables were expressed as relative and absolute frequencies and quantitative data as the mean (standard deviation, SD) when a normal distribution could be assumed or as the median and interquartile range when it was not possible. Normality was tested by the Shapiro–Wilk test.

The DR was the percentage of patients with successful detection of SLNs, defined as surgical removal of at least one lymph node visualized by lymphatic mapping with (99 mTc) Tc-nanocolloidal-albumin.

The pathologic findings in the SLNs were compared with the remaining axillary nodes to determine the FNR. A false negative event was defined as a case where SLNs did not show metastasis even though the residual disease was seen in other axillary nodes. The FNR was calculated as the number of false negative events divided by the total number of pathologically node-positive patients. In addition to the point estimate, 95% binomial confidence intervals (CIs) were calculated. All statistical analysis was performed with Stata/IC 13.0 for Windows.

## 3. Results

**Figure 1:**
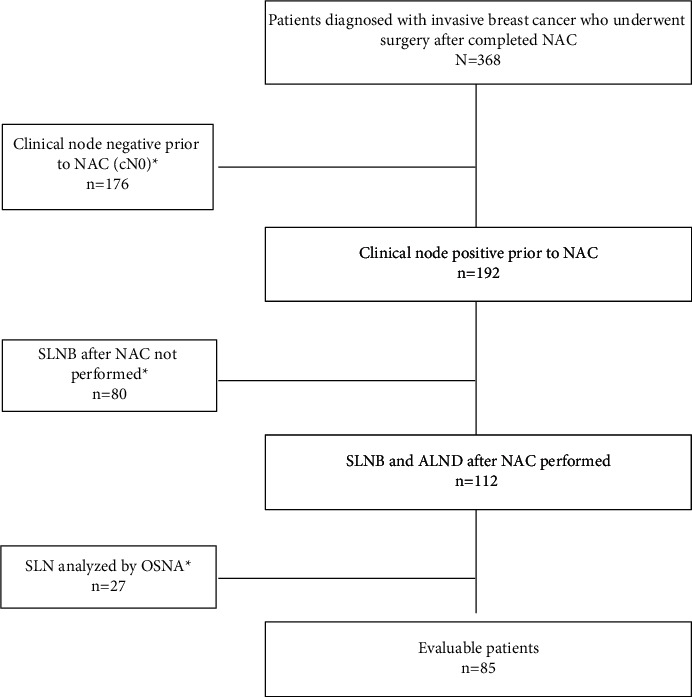
Flowchart of the study population and patient selection. NAC, neoadjuvant chemotherapy; SLNB, sentinel lymph node biopsy; SLN, sentinel lymph node; OSNA, one-step nucleic acid amplification; ALND, axillary lymph node dissection. The symbol ^*∗*^ indicates exclusion criteria that were as follows: clinically node negative prior to NAC (normal ultrasound and normal physical examination or/and negative fine needle aspiration); SLNB after NAC not performed due to cT4/cN2-3 or no complete axillary response after NAC; SLN analysed by OSNA.

A total of 368 patients with invasive breast cancer who underwent surgery after complete NAC were studied. Of them, 85 patients met the eligibility criteria and were enrolled in the study (Figure 1). Clinicopathologic features of all patients are summarized in Table 1. Systematic lymphadenectomy was performed in all patients, with an average of 10 lymph nodes removed (interquartile range (IQR):7; 13). Definitive pathological exam of all excised nodes (SLNB + ALND) showed a complete axillary response in 38 patients (44.7%) (Table 2).

**Figure 2:**
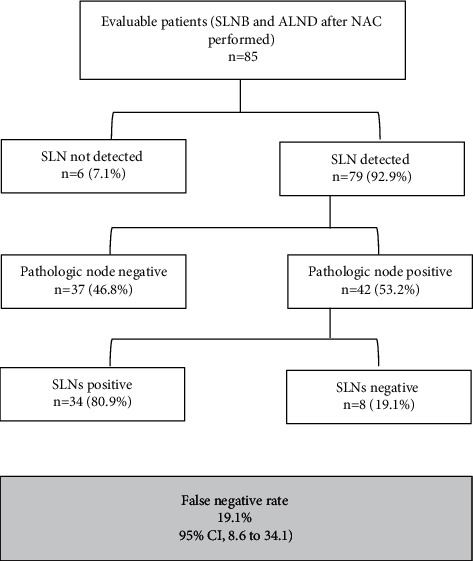
Ability of the pathologic evaluation of the sentinel lymph node dissection to predict nodal status. Sentinel lymph node biopsy, SLNB; axillary lymph node dissection, ALND; axillary pathological response post-NAC, ypN0.

Sentinel node mapping was successful in 79 patients (DR, 92.9%). Five of the six patients without migration of radiotracers had positive lymph nodes in ALND. The median number of SLNs removed was 3 (IQR 2; 5), and the median number of involved SLNs was 2 (IQR 1; 2). Stratifying by the number of SLNs removed, 17 (21.5%) patients had one SLN, 20 (25.3%) had two, and 42 (53.2%) had three or more (Figure 2 and Table 2).

As shown in Figure 2, 42 patients had positive lymph nodes in ALND and at least one SLN identified, and in eight of them, SLN was negative. Therefore, the FNR of SLNB was 19.1% (95% CI, 8.6–34.1). The SLN was the only positive node in 14 cases (33.3%). In patients with at least three SLNs identified and removed, there were two patients with a negative SLN but involved nodes in ALND, with an FNR of 8.7% (95% CI 1.1–28.4) (Table 3).

## 4. Discussion

The results of this study indicate that SLNB after NAC in patients with nodal involvement at diagnosis has an acceptable DR but a high FNR. In our study, the increase in the number of SLNs removed correlated with an improved FNR; however, the percentage of patients with at least three SLNs identified was not high. Considering that the presence of residual disease after NAC may indicate treatment resistance, optimizing its detection should be a priority to adequate adjuvant systemic and locoregional therapies [24–27].

SLNB after NAC is accurate for axillary staging in patients with clinically node-negative disease at diagnosis [3]. However, in node-positive patients, its performance has been controversial as per the results of the first prospective multicentric studies published in this matter [4, 5, 7]. The SENTINA trial included T1-3N1-2 tumours, and axillary staging was performed by physical examination and axillary ultrasound. The fine needle aspiration was not mandatory, but the clinical axillary response after NAC was demanded [5]. The ACOSOG study included T0-4N1-2 tumours, and the FNAC study included T1-3N1-2 disease [6, 7]; both trials required cytohistological confirmation of axillary involvement, while the clinical axillary response was not considered. In relation to the DR, which is defined as the percentage of patients with successful detection of SLNs by lymphatic mapping with (99 mTc) Tc-nanocolloidal-albumin, SENTINA, ACOSOG, and FNAC revealed values of 80.1%, 92.9%, and 87.6%, respectively. An improvement was achieved with the use of dual tracers in SENTINA and in ACOSOG studies to 87.8% and 93.8%, respectively. The adequate outcome of the DR (92.9%) observed in our series by only using 99 mTc may be related to the selection of patients with low breast and axillary burden (T1-3N1) and the superficial injection of the tracer [28–31].

Nonetheless, the FNR observed in our series was 19.1%, which is higher than recommended values of 10%. The SENTINA, ACOSOG 0071Z1071, and FNAC studies initially obtained an FNR of 14.2%, 12.6%, and 13.3%, respectively [4, 5, 7]. However, the accuracy of SLNB was closely related to the number of sentinel nodes removed. In the subgroup of women with at least three SLNs removed, the SENTINA and ACOSOG studies reported an FNR of 8.6% and 9.1%, respectively [4, 5]. Similarly, the FNR decreased to 8.7% in our series when at least three SLNs were excised. However, the percentage of patients with at least three SLNs was relatively low, ranging from 34% to 56.4% [4.5]. In our series, up to 53.2% of the patients had less than 3 SLNs resected, while half of them harboured a complete pathologic axillary response. Thus, considering only the number of resected lymph nodes may not benefit all patients. In fact, the use of only (99 mTc) Tc-nanocolloidal-albumin probably influenced the number of SLNs removed.

The use of selective SLNB only in patients with an axillary response after NAC, tested by axillary ultrasound (AUS), was proposed by ACOSOG to improve the FNR. However, the sensitivity of the axillary ultrasound ranges from 65% to 86%, meaning that the remaining disease will not be detected by this technique [8, 32]. Similarly, in our study, 47 out of 85 patients (55.3%) had axillary node involvement not detected by AUS.

Our findings reinforce the idea that additional techniques should be implemented to improve the FNR of SLNB after NAC. In this context, targeted lymph node biopsy (TLNB), TAD, and TAS have been proposed to improve axillary nodal staging and decrease the risk of remaining residual axillary disease [9–12, 33]. TLNB, firstly described by Donker et al., involves the selective removal of metastatic lymph nodes marked with an iodine seed prior to NAC. The reported FNR of the technique is 7% [33]. TAD includes removal of not only the SLN but also the lymph node known to contain metastases before chemotherapy, with a reported FNR lower than 10% in recent trials [9–11].

The contemporary concept of axillary surgery de-escalation includes TAS, which combines palpation-guided removal of suspicious nodes with the sentinel procedure and, optionally, imaging-guided localization. This technique has been evaluated in the TAXIS study, an international multicentre prospective randomized trial that reported an FNR of 1.8% with less radical axillary surgery [12].

Therefore, axillary staging after NAC in cN + patients at diagnosis remains unclear. This uncertainty is expressed in the heterogeneity of recommendations endorsed by different national and international societies, which range from SLNB to targeted axillary dissection (TAD) or ALND [13, 34, 35].

In addition, the efforts in de-escalating surgical procedures may be combined with adjusted, optimized, and individualized systemic treatments without a detrimental impact on the quality of life [36]. Updated results from the TAXIS and Alliance A11202 (ClinicalTrials.gov Identifier: NCT01901094) studies with disease-free and overall survival data are awaited.

The strength of our study was the strict standardization of conditions for the sentinel lymph node biopsy procedure and its performance by a multidisciplinary team specialized in breast cancer. The limitations are those inherently related to its retrospective design, the fact that the data could only be applied in selected patients (cT1-3cN1), and that the SLNB procedure was only performed with a single tracer. Globally, the results of the current study reinforce the available data from the literature.

## 5. Conclusions

SLNB after NAC in patients with nodal involvement has an adequate DR. Nonetheless, the FNR can be decreased to an acceptable range, improving its accuracy, when at least three SLNs are assessed. Our findings reinforce the idea that additional techniques should be implemented to improve the FNR of SLNB after NAC.

Further studies in this setting are required to determine the optimal axillary staging procedure without negatively impacting patients' outcomes and quality of life.

## Figures and Tables

**Table 1:** Clinicopathologic features.

Characteristics	Values
Age (years)	50.8 (12.0)
	50.0 (41.6; 58.3)
Postmenopausal	43 (50.6)
Histological type	
Ductal	80 (94.1)
Lobular	5 (5.9)
Histological Grade	
Grade 1	4 (4.7)
Grade 2	34 (40.0)
Grade 3	44 (51.8)
NR	3 (3.5)
Lymphovascular space invasion	10 (11.8)
NR	55 (64.7)
ICH subtype ^*∗*^	
Luminal A	9 (10.6)
Luminal B	21 (24.7)
Luminal /HER 2 positive	24 (28.2)
HER 2 enriched	15 (17.7)
Triple negative	16 (18.8)
T stage ^*∗∗*^	
T1a	0 (0.0)
T1b	3 (3.5)
T1c	6 (7.1)
T2	52 (61.2)
T3	24 (28.2)
Tumour size (mm)	42.8 (21.6)
	36 (28; 55)

Values expressed as *n* (%), mean (standard deviation), and p50 (p25; p75). NR, not reported; ICH, immunohistochemistry;  ^*∗*^Goldhirsch et al. and Viale et al; the symbol  ^*∗∗*^indicates the seventh edition of the American Joint Committee on Cancer (AJCC).

**Table 2:** Results of breast surgery, sentinel lymph node biopsy, and axillary lymph node dissection.

Breast surgery	—
Conservative	41 (48.2)
Mastectomy without reconstruction	9 (10.6)
Mastectomy with immediate reconstruction	34 (40.0)
Mastectomy and delayed reconstruction	1 (1.2)

Sentinel lymph node biopsy	
Sentinel lymph node migration	79 (92.9)
Number of sentinel lymph nodes removed	3.5 (2.3)
	3 (2; 5)
Patients with 1 sentinel lymph node removed	17 (21.5)
Patients with 2 sentinel lymph nodes removed	20 (25.3)
Patients with 3 or more lymph nodes removed	42 (53.2)
Number of positive sentinel lymph nodes	2.4 (2.3)
	2 (1; 2)
Final sentinel lymph node pathology report	
Negative	45 (57.0)
Isolated tumor cells	1 (1.3)
Micrometastases	4 (5.1)
Macrometastases	29 (36.7)

Axillary lymph node dissection	
Axillary lymphadenectomy	
Positive	52 (61.2)
Negative	33 (38.8)
Number of lymph nodes removed in lymphadenectomy	10.6 (5.5)
	10 (7;13)

Values expressed as n (%), mean (standard deviation), and p50 (p25; p75)

**Table 3:** Accuracy of the sentinel lymph node assessment for nodal metastases.

	Patients with at least one SLN removed, *N* = 79	Patients with at least three SLNs removed, *N* = 42
Sensitivity	81 (65.9–91.4) %	91.3 (72.0–98.9) %
Specificity	100.0 (90.5–100) %	100.0 (82.4–100) %
Negative predictive value	82.2 (67.9–92) %	90.5 (69.6–98.8) %
False negative rate	19.1 (8.6–34.1) %	8.7 (1.1–28.4) %

^*∗*^Values expressed in prevalence (95% confidence interval). SLN, sentinel lymph node. In all cases, *P* > 0.05.

## Data Availability

The datasets used to support the findings of this study are available from the corresponding author upon request.
